# Use of augmented and virtual reality in resuscitation training: A systematic review

**DOI:** 10.1016/j.resplu.2024.100643

**Published:** 2024-04-22

**Authors:** Adam Cheng, Nino Fijacko, Andrew Lockey, Robert Greif, Cristian Abelairas-Gomez, Lucija Gosak, Yiqun Lin, Education Implementation Team Task Force of the International Liaison Committee on Resuscitation (ILCOR), Natalie Anderson, Farhan Bhanji, Jan Breckwoldt, Andrea Cortegiani, Aaron Donoghue, Kathryn Eastwood, Barbara Farquharson, Ming-Ju Hiseih, Ying-Chih Ko, Elina Koota, Kasper G. Lauridsen, Tasuku Matsuyama, Sabine Nabecker, Kevin Nation, Alexander Olaussen, Taylor Sawyer, Sebastian Schnaubelt, Chih-Wei Yang, Joyce Yeung

**Affiliations:** a Department of Pediatrics and Emergency Medicine, Cumming School of Medicine, University of Calgary, KidSIM-ASPIRE Simulation Research Program, Alberta Children’s Hospital, Canada; b Faculty of Health Sciences, University of Maribor, Maribor University Medical Centre, Maribor, Slovenia; c Emergency Department, Calderdale & Huddersfield NHS Trust, Halifax, UK; d School of Human and Health Sciences, University of Huddersfield, Huddersfield, UK; e University of Bern, Bern, Switzerland; f School of Medicine, Sigmund Freud University Vienna, Vienna, Austria; g Faculty of Education Sciences and CLINURSID Research Group, Universidade de Santiago de Compostela, Santiago de Compostela, Spain; h Simulation and Intensive Care Unit of Santiago (SICRUS) Research Group, Health Research Institute of Santiago, University Hospital of Santiago de Compostela-CHUS, Santiago de Compostela, Spain; i Faculty of Health Sciences, University of Maribor, Maribor, Slovenia; j KidSIM-ASPIRE Simulation Research Program, Alberta Children’s Hospital, University of Calgary, Canada

**Keywords:** Resuscitation, Immersive Technology, Virtual Reality, Augmented Reality, Training, Life Support

## Abstract

**Objectives:**

To evaluate the effectiveness of augmented reality (AR) and virtual reality (VR), compared with other instructional methods, for basic and advanced life support training.

**Methods:**

This systematic review was part of the continuous evidence evaluation process of the International Liaison Committee on Resuscitation (ILCOR) and reported based on the Preferred Reporting Items for Systematic review and Meta-Analysis (PRISMA) guidelines and registered with PROSPERO (CRD42023376751). MEDLINE, EMBASE, and SCOPUS were searched from inception to January 16, 2024. We included all published studies comparing virtual or augmented reality to other methods of resuscitation training evaluating knowledge acquisition and retention, skills acquisition and retention, skill performance in real resuscitation, willingness to help, bystander CPR rate, and patients’ survival.

**Results:**

Our initial literature search identified 1807 citations. After removing duplicates, reviewing the titles and abstracts of the remaining 1301 articles, full text review of 74 articles and searching references lists of relevant articles, 19 studies were identified for analysis. AR was used in 4 studies to provide real-time feedback during CPR, demonstrating improved CPR performance compared to groups trained with no feedback, but no difference when compared to other sources of CPR feedback. VR use in resuscitation training was explored in 15 studies, with the majority of studies that assessed CPR skills favoring other interventions over VR, or showing no difference between groups.

**Conclusion:**

Augmented and virtual reality can be used to support resuscitation training of lay people and healthcare professionals, however current evidence does not clearly demonstrate a consistent benefit when compared to other methods of training.

## Introduction

Cardiopulmonary arrest is a challenging and critical healthcare problem associated with poor survival rates from in and out-of-hospital events.^1^, ^2^ Based on the formula for survival, improving survival outcomes from cardiopulmonary arrest is dependent upon advancements in medical science, educational efficiency, and local implementation.^3^ Resuscitation training for basic life support (BLS) and advanced life support (ALS) improves healthcare professional knowledge and skill acquisition, however consistent transfer of these skills to real-life resuscitation remains a challenge.^4^, ^5^ Enhancing learning and performance outcomes from resuscitation training requires thoughtful integration of instructional methods with novel technology. Immersive technology, such as augmented reality (AR) and virtual reality (VR) offers a promising new way to deliver resuscitation training to lay people and healthcare professionals.^4^, ^6^, ^7^, ^8^, ^9^, ^10^

Augmented reality is comprised of a wearable device generating a holographic image overlaid into the real clinical environment, permitting the user to interact with the hologram and objects in the real environment in an integrated fashion.^8^, ^11^. Studies have explored the use of AR in providing visual cues and prompting during technical skills such as cardiopulmonary resuscitation (CPR) and patient care.^11^, ^12^, ^13^, ^14^ Virtual reality is defined as a “three dimensional computer-generated simulated space, which attempts to replicate real world or imaginary environments and interactions”.^8^ VR enviroments allow users to engage with simulated patients within immersive and interactive scenarios, without integration of objects in the real environment. International resuscitation guidelines and consensus statements have called for more research to advance our knowledge of AR and VR use during resuscitation training.^4^, ^5^

Recent reviews of the AR and VR healthcare literature described the potential applicability of immersive technology in the education and training of healthcare professionals.^7^, ^9^, ^10^. Immersive technology been applied across a variety of different medical fields to train healthcare professionals, with the advantages of realism, replayability, and time-effectiveness.^7^, ^9^ The value of immersive technology for basic and advanced life support training of lay persons and healthcare professionals is unclear. Clarifying the value of AR and VR-based training will provide importance guidance for resuscitation training programs and global resuscitation councils. In this systematic review, we aim to describe if using virtual or augmented reality, compared with other methods of basic and advanced life support training, improves knowledge acquisition and retention, skill acquisition and retention, skill performance during real resuscitation, willingness to help, bystander CPR rates, and patient survival rates.

## Methods

### Eligibility criteria

This sytematic review was conducted by the Education, Implementation and Teams (EIT) Task Force of the International Liaison Committee on Resuscitation (ILCOR) as part of the continuous evidence evaluation process of resuscitation literature informing international consensus treatment recommendations.^15^, ^16^ The review was conducted and reported in compliance with the Preferred Reporting Items for Systematic Review and Meta-Analysis (PRISMA) guidelines,^17^ and registered with the Prospective Registry for Systematic Reviews (PROSPERO CRD42023376751, protocol available at https://www.crd.york.ac.uk/prospero/display_record.php?IS=CRD42023376751). The research question was structured using the ‘PICOST’ (Population, Intervention, Comparison, Outcome, Study Design, Timeframe) format as per ILCOR evidence reviews:

**P**opulation: All laypersons and healthcare professionals (including healthcare trainees) in any educational setting;
**I**ntervention: Immersive technologies (e.g. AR, VR) as part of the instructional design to train neonatal, pediatric, and adult basic and advanced life support;
**C**omparison: Other methods of resuscitation training in basic and advanced life support (e.g., traditional manikin-based simulation training, other);
**O**utcomes: Knowledge acquisition and retention, skills acquisition and retention (i.e. CPR quality), skill performance in real resuscitation (i.e. CPR quality), willingness to help, bystander CPR rate, and patients’ survival;
**S**tudy Design: Randomized controlled trials (RCTs) and non-randomized studies (non-randomized controlled trials, interrupted time series, controlled before-and-after studies, cohort studies and case series where *n* > 5, conference abstracts) and research letters were eligible for inclusion;
**T**imeframe: Inception to January 16, 2024.

All relevant publications in any language were included as long as there was an English abstract available.

### Definitions

For the purposes of this systematic review, we defined AR as a computer-generated holographic image overlaid into the real clinical environment, permitting the user to interact with the hologram and objects in the real environment in an integrated fashion,^8^, ^11^ and VR as a “three dimensional computer-generated simulated space, which attempts to replicate real world or imaginary environments and interactions”.^8^

### Information sources and search strategy

We utilized a search strategy developed in conjunction with an information specialist using (but not limited to) the following key terms: “cardiopulmonary resuscitation”, “basic life support”, “advanced life support”, “cardiac arrest”, “chest compressions”, “augmented reality”, “virtual reality”, “VR sim”, “VR/AR”, “virtual scenarios’ and “mixed reality”. The detailed search strategy is shown in online supplementary material. We searched Medline, Embase, and Scopus from inception until January 16, 2024. Grey literature was not searched. Reference lists of identified studies and review articles were scanned to identify additional relevant publications.

### Study selection

Duplicates were detected using Rayyan (a web-based software for systematic reviews), with one reviewer (YL) screening all duplicates and removing them when appropriate. Three pairs of reviewers independently (AC,YL; NF,CAG; RG,AL) screened titles and abstracts using Rayyan, excluding all papers that did not meet eligibility criteria. Reviewer pairs resolved disagreements via discussion to reach a consensus. In the rare instance when a consensus was not reached, full text of the paper was obtained for review. The full text of remaining papers were independently reviewed for eligibility by three pairs of reviewers. The remaining disagreements were discussed amongst reviewer pairs to reach consensus on the final group of articles.

### Data extraction

After identification of the final group of articles, two reviewers (YL, AC) independently extracted relevant data from all the relevant articles into an Excel spreadsheet. Extracted data was double checked and differences were resolved through discussion. Data extracted included author, publication year, country, study design, population, sample size, intervention and comparison, outcome measures, and results.

### Risk of bias assessment

Two pairs of reviewers independently assessed the included papers for risk of bias using two tools: the Risk of Bias 2 (RoB 2) tool was used for RCTs,^18^ and the Risk of Bias in Non-randomised Studies of Intervnetions (ROBINS-I) tool was used for non-RCTs.^19^ Disagreements between reviewers was resolved by discussion to reach consensus.

### Synthesis of results

The overall certainty of evidence was assessed using the Grading of Recommendations Assessment, Development and Evaluation (GRADE) methodology. We elected not to conduct a meta-analysis due to significant heterogeneity in methodology (e.g. intervention type, control groups, study populations) and outcome measures. Results were reported in compliance with the Synthesis without meta-analysis (SWiM) reporting guidelines for systematic reviews.^20^ EIT task force members discussed the extracted data and results tables on several virtual conference calls to craft treatment recommendations and identify key insights and future opportunities for research.

## Results

### Study characteristics

**Fig. 1:**
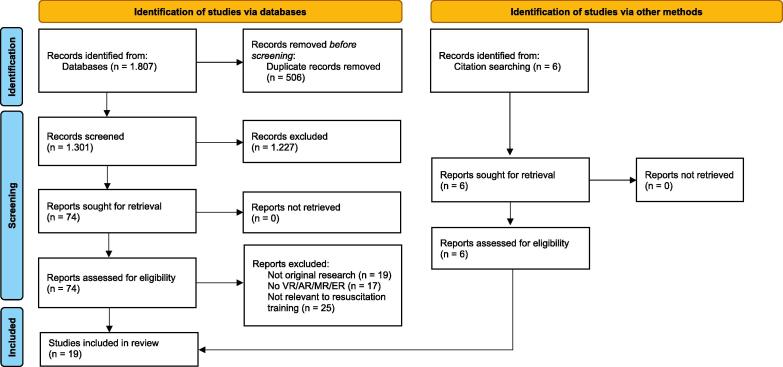
PRISMA Diagram.

Our initial literature search identified 1807 citations. After removing 506 duplicates, 1301 articles were screened by reviewing the titles and abstracts (Fig. 1). Of these, 74 articles remained for full-text review, of which 13 studies were selected for inclusion. An additional 6 studies were identified via search of references lists of relevant articles, with a total of 19 studies included in the final analysis with publication years ranging from 2014 to 2023 (Table 1).^11^, ^12^, ^13^, ^21^, ^22^, ^23^, ^24^, ^25^, ^26^, ^27^, ^28^, ^29^, ^30^, ^31^, ^32^, ^33^ Seventeen of these studies were randomized controlled trials^11^, ^12^, ^13^, ^14^, ^21^, ^22^, ^23^, ^24^, ^25^, ^26^, ^27^, ^28^, ^29^, ^30^, ^31^, ^32^, ^33^, ^34^ and two were a non-randomized controlled trials.^29^, ^35^

**Table 1:** Overview of augmented reality and virtual reality studies.

Author & Year Published	Study Type	Study Population & Sample Size	Intervention Group	Control Group	Conclusion
Augmented Reality Studies					
Leary et al. 2020^11^	RCT	Nursing students *N* = 100	AR-assisted feedback	CPR manikin with regular audiovisual feedback	Favors non-AR (regular AV feedback, not significant)
Hou et al. 2022 (a)^13^	RCT	Lay providers *N* = 28	Real-time AR-assisted CPR training	Conventional real-time supervisor-assisted CPR training	Non-significant (Favors AR-assisted instruction)
Hou et al. 2022 (b)^14^	RCT	Lay providers *N* = 163	Real-time AR-assisted self-guided CPR training	Conventional instructor-led CPR training	Non significant difference between groups
Jeffers et al. 2022^12^	RCT	Healthcare providers and students *N* = 34	2-min CPR with AR-assisted feedback	2-min CPR on manikin with no feedback	Favors AR
Virtual Reality (VR) Training for Basic Life Support (Lay People)					
Leary et al. 2019^30^	RCT	Adult Lay rescuers *N* = 10	CPR training with VR mobile App	PR training with mobile App (2D)	Favors non-VR in CPR quality
Barsom et al. 2020^31^	RCT	High school students *N* = 40	e-learning module + VR training	e-learning module + 2D video	Favors VR
Nas et al. 2020^22^	RCT	Adult lay people *N* = 381	20 min VR-based CPR training	Instructor-led 20 min CPR training	Favors non-VR (Face-to-face training)
Liu et al. 2021^25^	Quasi-experimental, 2x2 factorial	1st year college students *N* = 120	Intervention 1: VR training without pre-training intervention Intervention 2: VR training with pre-training intervention	Control 1: video training without pre-training intervention Control 2: Video training with pre-training intervention	Non significant difference between groups
Hubail et al. 2022^23^	RCT	Adult lay providers *N* = 26	Instructor led training, participants with VR headsets and hand sensors	4-hour Certified instructor-led course with lectures and hands-on skill practice	Favors non-VR (non-significant)
Liu et al. 2022^26^	Quasi-experimental	Kindergarten teachers *N* = 50	VR-based CPR and AED training	Conventional video-based training	Favors VR
Nas et al. 2022^21^	Secondary analysis of data from RCT	Adult lay people *N* = 188	20 min VR-based CPR training	Instructor-led 20 min CPR training	Favors non-VR (Face-to-face training)
Castillo et al. 2023^24^	Quasi-experimental	1st year university students *N* = 341	Training with Virtual Reality	Traditional Training	Non significant difference between groups
Chang et al. 2023^34^	RCT *N* = 75	Undergraduate and postgraduate students *N* = 75	Training with virtual reality	Control 1: Face to face lecture + practice Control 2: Hybrid: Video + practice	Favors non-VR
Kim et al. 2023^35^	Quasi-experimental	Firefighters *N* = 121	Virtual Reality Training	Flipped Classroom Training	Favors VR
Virtual Reality (VR) for Basic Life Support Training (Healthcare Providers)					
Aksoy et al. 2019^27^	RCT	Paramedic students *N* = 50	VR-based serious game for BLS knowledge	PC-tablet serious game for BLS knowledge	Favors VR
Issleib et al 2021^32^	RCT	First-year medical students *N* = 160	25 min VR module + 10 min VR module chest compression	Conventional BLS course with seminar and basic skill training	Favors non-VR (conventional teaching)
Moll-Khosrawi et al. 2022^33^	RCT	First-year medical students *N* = 88	Web-based BLS training + VR BLS training module	Web-based BLS training	Favors VR
Virtual Reality (VR) for Advanced Life Support Training (Healthcare Providers)					
Umoren et al. 2021^28^	RCT	Practicing nurses and midwives *N* = 274	Intervention 1: video + HBB providers’ guide Intervention2 (VR): VR + digital HBB provider’s guide	Standard practice, Digital HBB provider’s guide	Non significant difference between groups
Yang et al. 2022^29^	Quasi-experimental	Nursing students *N* = 74	NRP gamification VR program	Control Group: Online NRP program lecture Simulation Group: NRP training with high fidelity simulator	Non significant difference between groups

*Abbreviations:* ACLS – advanced cardiac life support, AED – automated external defibrillator, AR – augmented reality, BLS – basic life support, CPR − cardiopulmonary resuscitation, HBB – helping babies breathe, HCP − health care professional, NRP – neonatal resuscitation program, RCT – randomized controlled trial, VR – virtual reality.

Four studies examined the use of augmented reality in BLS training, with all studies using AR to provide real-time CPR feedback.^11^, ^12^, ^13^, ^14^ Thirteen studies explored the use of VR for BLS training, with ten studies assessing use amongst lay people^21^, ^22^, ^23^, ^24^, ^25^, ^26^, ^30^, ^31^, ^34^, ^35^ (Table 1) and three studies evaluating VR use in healthcare professionals^27^, ^32^, ^33^ (Table 1). Amongst these studies, intervention groups all featured VR as the primary instructional methodology, either alone^21^, ^22^, ^23^, ^24^, ^25^, ^26^, ^30^, ^34^, ^35^ or in combination with other features such as a provider’s guide or training module^31^, ^32^, ^33^ or gamification.^27^ Control groups were highly variable, and included: instructor-led training,^21^, ^22^, ^23^, ^24^, ^32^ video or web-based training,^25^, ^26^, ^31^, ^33^ mobile-app based training,^30^ or a tablet-based serious game.^27^ Two studies described the use of VR for ALS training^28^, ^29^ (Table 1). One study compared VR supplemented by a provider’s guide to standard training and video-based training with the provider’s guide,^28^ and the other study compared gamified VR training to instructor-led neonatal resuscitation program training using high fidelity simulation.^29^ No studies reported skill performance (i.e. CPR quality) in real patients, patient survival outcomes or bystander CPR rates. Risk of bias assessment for individual studies varied from low to high (Table 2, Table 3). Overall certainty of evidence was rated as very low and downgraded due to risk of bias, indirectness and inconsistency.

**Table 2:** Risk of bias assessment for randomized controlled trials.

First author, year	Type of course	Type of participant	Type of immersive techno-logy	Randomization	Deviations from intended intervention	Outcome data missing	Measurement of outcome	Selection of reported results	Overall
Aksoy, 2019^27^	BLS	HCP	VR	High	Low	Some	Low	Low	High
Leary, 2019^30^	BLS	Lay rescuer	VR	High	Low	Low	Low	Low	High
Barsom, 2020^31^	BLS	Lay rescuer	VR	High	Low	Low	Low	Low	High
Leary, 2020^11^	BLS	HCP	AR	Low	Low	Low	Low	Low	Low
Nas, 2020^22^	BLS	Lay rescuer	VR	Low	Low	Low	Low	Low	Low
Issleib, 2021^32^	BLS	HCP	VR	High	Some	Some	Low	Low	High
Liu, 2021^25^	BLS	Lay rescuer	VR	High	Low	Low	Low	Low	High
Umoren, 2021^28^	ALS	HCP	VR	Low	Low	Low	Low	Low	Low
Hou, 2022 (a)^13^	BLS	Lay rescuer	AR	Low	Low	Low	Low	Low	Low
Hou, 2022 (b)^13^	BLS	Lay rescuer	AR	Low	Low	Low	Low	Low	Low
Hubail, 2022^23^	BLS	Lay rescuer	VR	Low	Low	Low	Low	Low	Low
Jeffers, 2022^12^	PALS	HCP	AR	Low	Low	Low	High	Low	High
Liu, 2022^26^	BLS	Lay rescuer	VR	High	Low	Low	Low	Low	High
Moll Khosrawi, 2022^33^	BLS	HCP	VR	Low	Low	Low	Low	Low	Low
Nas, 2022^21^	BLS	Lay rescuer	VR	Low	Low	High	High	Low	High
Castillo, 2023^24^	BLS	Lay rescuer	VR	High	Low	Some	Low	Low	High
Chang, 2023^34^	BLS	Lay rescuer	VR	High	Low	Some	High	Low	High

*Abbreviations:* ALS – Advanced Life Support, AR – Augmented reality, BLS – Basic Life Support, HCP – Health Care Professional, VR – Virtual reality.

**Table 3:** Risk of bias assessment for non-randomized controlled trials.

Study	Type of training	Type of Participant	Type of immersive technology	Confound-ing	Selection	Classification of intervention	Deviations from intended intervention	Outcome data missing	Measure-ment of outcomes	Selection of reported results	Overall
Yang, 2022^29^	ALS	HCP	VR	Serious	Moderate	Low	Low	Low	Low	Low	Serious
Kim, 2023^35^	BLS	Lay rescuer	VR	Serious	Moderate	Low	Low	Low	Low	Low	Serious

*Abbreviations:* ALS – Advanced Life Support, BLS – Basic Life Support, HCP – Health Care Professional, VR – Virtual reality.

### Augmented reality – CPR skill outcomes

#### CPR depth, rate and overall CPR performance

Three studies reported CPR depth performance with and without use of AR-based CPR feedback during training, with all demonstrating no significant difference in CPR depth performance between the intervention group and the control groups that received other forms of CPR feedback or guidance from instructors (Table 4).^11^, ^13^, ^14^ Two studies assessed CPR depth compliance after training, with one reporting improved CPR depth compliance in the AR group,^12^ and the other showing no difference between groups.^14^ Three studies evaluating CPR rate immediately after training found no significant difference in CPR rate performance between control and intervention groups^11^, ^13^, ^14^; two of these studies included control arm groups that received CPR feedback from other sources.^11^, ^13^ Two studies found no significant difference in CPR rate compliance after training when comparing participants trained with and without AR-assisted feedback.^12^, ^14^ Two studies assessed overall CPR performance with mixed results. One study found significantly improved overall CPR performance in the AR group,^12^ while the other study found significantly better overall CPR performance in the control group (CPR manikin with regular audiovisual feedback system).^11^

**Table 4:** Outcomes for Augmented Reality (AR) Studies.

Study	Number – Control vs. Intervention (AR)	Outcome – Control	Outcome – Intervention (AR)	*P* value
CPR Depth				
Leary 2020^11^	50 vs. 50; Total 100	49 mm	52 mm	*P* = 0.09
Hou 2022(a)^13^	13 vs. 14; Total 27	48.7 mm	50.5 mm	*P* = 0.32
Hou 2022(b)^14^	81 vs. 82 Total 163	55.5 mm	55.9 mm	*P* = NS
CPR Depth Compliance				
Jeffers 2022^12^	18 vs. 16; Total 34	21%	79%	*P* < 0.01
Hou 2022(b)^14^	81 vs. 82 Total 163	73%	66%	*P* = 0.33
CPR Rate				
Leary 2020^11^	50 vs. 50; Total 100	117 cpm	122 cpm	*P* = 0.10
Hou 2022(a)^13^	13 vs. 14; Total 27	110 cpm	109 cpm	*P* = 0.48
Hou 2022(b)^14^	81 vs. 82 Total 163	111 cpm	109 cpm	*P* = NS
CPR Rate Compliance				
Jeffers 2022^12^	18 vs. 16; Total 34	76%	90%	*P* = 0.06
Hou 2022(b)^14^	81 vs. 82 Total 163	99%	99%	*P* = NS
Overall CPR Performance				
Leary 2020^11^	50 vs. 50; Total 100	36%	16%	*P* = 0.03
Jeffers 2022^12^	18 vs. 16; Total 34	17%	73%	*P* < 0.01

*Abbreviations:* AR – augmented reality, cpm – compressions per minute, NS – not significant.

### Virtual reality – BLS knowledge, CPR skills and willingness to perform CPR

#### Knowledge acquisition and retention

In four studies there were significantly higher knowledge scores with VR training compared to other forms of non-VR training, such as a PC-tablet based serious game,^27^ an e-learning module with video,^31^ video-based training,^26^ and flipped classroom training^35^ (Table 5). Two studies showed no difference in participant knowledge when comparing VR training to traditional training^24^ or video-based training,^25^ and one study showed improved knowledge scores with non-VR based training methods.^34^ Amongst the three studies evaluating knowledge retention, one study demonstrated improved knowledge retention at 5 weeks post-training in the virtual reality group,^26^ while the other two studies showed no difference in knowledge retention at 6 months.^21^, ^24^

**Table 5:** Knowledge Outcomes for Virtual Reality (VR) BLS studies.

Study	Number – Control vs. Intervention (VR)	Outcome – Control	Outcome – Intervention (VR)	*P* value
Knowledge acquisition				
Aksoy 2019^27^	18 vs. 22; Total 40	Mean 8.9 (pre-post difference in knowledge test score)	Mean 17.6 (pre-post difference in knowledge test score)	*P* = 0.021
Barsom 2020^31^	20 vs. 20; Total 40	Median 25 (pre-post difference in knowledge score)	Median 32 (pre-post difference in knowledge score)	*P* = 0.035
Liu 2021^25^	30 vs. 30 (video vs. VR; both without pretraining); Total 60	6.53 (score after training)	6.43 (score after training)	*P* = 0.66
Liu 2022^26^	25 vs. 25; Total 50	2.24 (pre-post difference in knowledge score)	3.32 (pre-post difference in knowledge score)	*P* = 0.03
Castillo 2023^24^	116 vs. 125; Total 241	8.21 (score after training)	8.44 (score after training)	*P* = 0.24
Chang 2023^34^	23 vs. 30 vs. 22 Total 75	89.2 (control 1) and 88.2 (control 2) (score after training)	81.2 (score after training)	*P* < 0.05
Kim 2023^35^	61 vs. 60; Total 121	12.33 (score after training)	15.33 (score after training)	*P* < 0.01
Knowledge retention				
Liu 2022^26^	25 vs. 25; Total 50	−0.08 (pre-post difference in knowledge score at 5 weeks)	1.84 (pre-post difference in knowledge score at 5 weeks)	*P* = 0.02
Nas 2022^21^	97 vs. 91; Total 188	7 (score at 6 months)	7 (score at 6 months)	*P* = 0.81
Castillo 2023^24^	56 vs. 64; Total 120	6.55 (score at 6 months)	6.25 (score at 6 months)	*P* = 0.75

*Abbreviations:* VR – virtual reality.

#### CPR depth, rate, chest recoil and overall CPR performance

Of the four studies that reported CPR depth performance after training, two demonstrated significantly better CPR depth in the control group compared to those who received virtual reality training,^22^, ^30^ and the other two studies demonstrated no significant difference in CPR depth performance between groups (Table 6).^23^, ^24^ The two studies that assessed CPR depth compliance after training found that participants in the non-VR training groups had significantly better CPR depth compliance compared to those who received VR training.^22^, ^34^ Three studies evaluated CPR rate immediate after training, with one study reporting higher CPR rates in the intervention group,^22^ and the other two studies describing no difference in CPR rate performance between groups.^23^, ^30^ For the outcome of CPR rate compliance, three studies reported mixed results, with two studies showing significantly improved rate compliance in the non-VR control groups,^22^, ^34^ and the other study showing no difference between groups.^24^ Four studies evaluated chest recoil compliance after training, with three studies demonstrating no difference between groups,^23^, ^24^, ^34^ and one study reported better chest recoil compliance amongst those who received virtual reality training.^22^ For the outcome of overall CPR performance (i.e. CPR scores) after training, one study found improved CPR scores in the VR training group,^35^ another found non-VR training to be superior,^34^ and two studies found no difference in scores when comparing virtual reality training to instructor-led training with lectures^23^ and video-based training.^25^ Only one study measured retention of CPR skills 6 months after training, reporting no difference in CPR depth, rate, or chest recoil performance at 6 months between those who received traditional training and those trained using virtual reality.^24^

**Table 6:** Skills Outcomes for Virtual Reality (VR) BLS studies.

Study	Number – Control vs. Intervention (VR)	Outcome – Control	Outcome – Intervention (VR)	*P* value
No Flow Time/Chest Compression Fraction				
Nas 2020^22^	177 vs. 175; Total 352	67% (CCF)	61%	*P* < 0.01
Issleib 2021^32^	104 vs. 56; Total 160	82sec (no flow time)	93sec	*P* < 0.01
Moll Khosrawi 2022^33^	42 vs. 46; Total 88	8.0sec (no flow time)	5.8sec	*P* = 0.01
CPR Depth				
Leary 2019^30^	53 vs. 52; Total 105	44.0 mm	38.0 mm	*P* = 0.05
Nas 2020^22^	177 vs. 175; Total 352	56.8 mm	49.1 mm	*P* < 0.01
Hubail 2022^23^	13 vs. 13; Total 26	47.2 mm	45.1 mm	*P* = 0.21
Castillo 2023^24^	116 vs. 125; Total 241	47.1 mm	46.0 mm	*P* = 0.24
CPR Depth Compliance				
Nas 2020^22^	177 vs. 175; Total 352	75%	51%	*P* < 0.01
Chang 2023^34^	23 (CG1) vs. 30 (CG2) vs. 22 Total 75	99.8% (CG1) and 98.3% (CG2)	89.5%	*P* < 0.05
CPR Rate				
Leary 2019^30^	53 vs. 52; Total 105	112 bpm	104 bpm	*P* = NS
Nas 2020^22^	177 vs. 175; Total 352	108 bpm	114 bpm	*P* < 0.01
Hubail 2022^23^	13 vs. 13; Total 26	114 bpm	111 bpm	*P* = 0.36
CPR Rate Compliance				
Nas 2020^22^	177 vs. 175; Total 352	63%	50%	*P* = 0.01
Castillo 2023^24^	116 vs. 125; Total 241	61.9%	60.3%	*P* = 0.71
Chang 2023^34^	23 (CG1) vs. 30 (CG2) vs. 22 Total 75	93% (CG1) and 94.5% (CG2)	49.6%	*P* < 0.05
Chest Recoil Compliance				
Nas 2020^22^	177 vs. 175; Total 352	88%	98%	*P* = 0.02
Hubail 2022^23^	13 vs. 13; Total 26	78.2%	83.4%	*P* = 0.33
Castillo 2023^24^	116 vs. 125; Total 241	70.5%	71.6%	*P* = 0.80
Chang 2023^34^	23 (CG1) vs. 30 (CG2) vs. 22 Total 75	91.8% (CG1) and 82.8% (CG2)	83.8%	*P* = NS
Overall CPR Performance				
Liu 2021^25^	30 vs. 30 (video vs. VR; both without pretraining); Total 60	66.9 (CPR score after training)	53.7 (CPR score after training)	*P* = 0.82
Hubail 2022^23^	13 vs. 13; Total 26	9.61 (CPR Score after training)	8.53 (CPR Score after training)	*P* = 0.09
Chang 2023^34^	23 (CG1) vs. 30 (CG2) vs. 22; Total 75	94.7 (CG1) and 93.6 (CG2) (CPR Score after training)	83.6 (CPR Score after training)	*P* < 0.05
Kim 2023^35^	61 vs. 60; Total 121	76.9 (CPR Score after training)	85.9 (CPR Score after training)	*P* < 0.01
CPR Depth – Retention at 6 months				
Castillo 2023^24^	56 vs. 64; Total 120	44.7 mm	42.7 mm	*P* = 0.33
CPR Rate Compliance – Retention at 6 months				
Castillo 2023^24^	56 vs. 64 Total 120	52.2%	50.1%	*P* = 0.86
Chest Recoil Compliance − Retention				
Castillo 2023^24^	56 vs. 64; Total 120	79.5%	77.3%	*P* = 0.57

*Abbreviations:* CCF – chest compression fraction, CG – control group, CPR – cardiopulmonary resuscitation, NS – not significant, VR – virtual reality.

#### Willingness to perform CPR

One study recruited adult lay people to instructor-led CPR training or VR-based CPR training, and found that those who received instructor-led CPR training were more willing to perform CPR at 6 months post-training.^21^

### Virtual reality – ALS knowledge and clinical performance

#### Knowledge

One study with nursing students as participants compared neonatal resuscitation program (NRP) with a high fidelity simulator to NRP training with virtual reality and showed no significant difference in knowledge immediately post-training^29^ (Table 7).

**Table 7:** Outcomes for Virtual Reality (VR) ALS studies.

Study	Number – Control vs. Intervention (VR)	Outcome – Control	Outcome – Intervention (VR)	*P* value
Knowledge				
Yang 2022^29^	28 vs. 29	3.00 (pre-post difference)	5.48 (pre-post difference)	*P* = NS
Clinical Performance – (OSCE A test)				
Umoren 2021^28^	88 vs. 91	72% (post training)	76% (post training)	*P* = 0.63
Umoren 2021^28^	86 vs. 87	72% (retention at 6 months)	76% (retention at 6 months)	*P* = 0.61

*Abbreviations:* ALS – Advanced Life Support, OSCE – Objective Structured Clinical Exam, VR – virtual reality.

#### Clinical performance

One study comparing standard Helping Babies Breathe (HBB) training to VR-based HBB training found no significant difference in test scores between groups immediately post training and at 6 months post training^28^ (Table 7).

## Discussion

Our systematic review exploring the value of immersive technology in resuscitation training identified 19 studies that described different applications of AR and VR for basic and advanced life support training. Augmented reality was used to provide real-time feedback during CPR, demonstrating improved CPR performance compared to groups trained with no feedback^12^; but no significant difference when compared with groups receiving feedback from a CPR feedback system^11^ or an instructor.^13^, ^14^ The use of VR in resuscitation training showed mixed results for knowledge acquisition and retention, while the majority of studies assessing CPR skills showed no difference between VR and control groups or favored other interventions over VR.^22^, ^23^, ^24^, ^25^, ^29^, ^30^, ^32^, ^34^

Augmented reality has seen expanded use in healthcare, with applications to support clinical care delivery and education of front-line healthcare professionals.^6^, ^10^, ^36^ With AR, users are provided with ‘powerful, contextual and situated learning experiences as well as construct new understanding based upon user’s interactions’^10^ with virtual objects and those in the clinical environment. Our review identified four studies which utilized these features of AR to facilitate the delivery of CPR feedback during training.^11^, ^12^, ^13^, ^14^ These results are perhaps not so surprising, supporting the notion that CPR feedback during training improves performance,^4^, ^37^ whilst concurrently highlighting that AR-based CPR feedback was not superior over other sources of feedback (e.g. CPR feedback device or instructor). Prior studies have illustrated how AR can be effectively used to support procedural skills training (e.g. bedside ultrasound, central line insertion)^6^, ^10^ and provide decision support and clinical prompts during actual resuscitative care.^38^, ^39^ We see these as exciting avenues for future resuscitation education research, where AR could potentially be used to improve acquisition of key procedural skills other than CPR, such as intubation, intraosseous needle insertion, and defibrillation. AR could also potentially be used to provide expert guidance via clinical prompts during resuscitation training, helping to reinforce quick and efficient decision making during cardiac arrest cases. Real-time integration of data from patient monitors and other medical devices (e.g. CPR feedback defibrillator) into the AR interface could streamline and personalize data delivery to healthcare professionals to enhance care. Future studies could explore how data-driven, AR-based clinical decision support during training affects individual and team-based performance during patient care.

Virtual reality provides users with an immersive learning experience within a computer-generated three dimensional environment.^7^ Within this virtual clinical environment, users have opportunity to apply clinical reasoning and decision making during simulated scenarios. Prior reviews of the VR literature in emergency medicine and healthcare simulation report mixed results as it relates to VR’s impact on knowledge acquisition when compared to other educational modalities (e.g. manikin-based simulation, video-based learning, e-learning, etc.).^6^, ^7^, ^9^ Our review yielded similarly mixed results for acquisition of resuscitation knowledge, as VR studies were highly heterogenous with respect to type of VR hardware, amount of exposure to virtual cases, clinical case complexity, degree of gamification and interactivity, timing of feedback, and nature of debriefing. Few studies took opportunity to conduct a full debriefing after the VR simulation, representing a missed opportunity to help consolidate learning. Blending VR with other evidence-based instructional design features, such as feedback, debriefing, spaced learning, or deliberate practice may help to unlock the potential of immersive technology for resuscitation training.^5^

In contrast to AR, VR technology does not ‘allow overlaying of computer-generated images onto a real-life viewing window’^8^ with seamless integration of real-life objects in the display. This represents a possible disadvantage when using VR for CPR skills training. Amongst the VR studies identified in this review, a variety of different alternatives were used for CPR training in lieu of traditional CPR manikins or torsos, including pillows,^22^, ^25^ stacking VR controllers on top of each other,^23^, ^26^ or pressing a chest compression button within the VR interface.^30^ These objects and approaches lack the ability to simulate chest wall compliance and the forces required to deliver effective CPR, potentially explaining why VR was not superior to other instructional methods for CPR skills training. Future attempts to utilize VR for resuscitation skills training consider whether VR can be blended with other training tools to provide the features, functionality, and feedback necessary to appropriately engage learners with the psychomotor behaviours required to effectively perform the procedural skill.

### Limitations, knowledge gaps, and future research

Our review has several limitations. While our review identified 19 relevant studies, the heterogeneity with respect to the design of the intervention (i.e. application of AR or VR), comparison group, and participant type made meta-analysis undesirable. Many of the studies reported CPR outcomes, but the CPR metrics reported were also highly variable (eg. CPR depth vs. CPR depth compliance vs. Overall CPR performance), thus precluding our ability to pool results across relevant studies. These limitations made it difficult to determine the true value of immersive technology across different contexts (i.e. basic vs. advanced life support) and learner groups (i.e. lay people vs. healthcare professionals). We acknowledge that our study was bounded by our definitions of AR and VR – broader or different definitions may have resulted in different outcomes. As our study was focused only on use of AR and VR during resuscitation training, we did not review literature that explored the application of immersive technology in non-training clinical environments. To further advance the implementation of immersive technology in resuscitation education, we encourage researchers to conduct research that: (1) explores the relative and synergistic effect of immersive technology when combined with other educational strategies; and (2) clearly delineates the impact on short and long term term retention of knowledge and skills..

## Conclusion

Augmented and virtual reality can be used to support resuscitation training of lay people and healthcare professionals, however current evidence does not clearly demonstrate a consistent benefit when compared to other methods of basic and advanced life support training.

## Funding

This work has been supported using public funds via the American Heart Association.

## CRediT authorship contribution statement

**Adam Cheng:** Writing – review & editing, Writing – original draft, Supervision, Methodology, Formal analysis, Data curation, Conceptualization. **Nino Fijacko:** Writing – review & editing, Methodology, Data curation, Conceptualization. **Andrew Lockey:** Writing – review & editing, Methodology, Formal analysis, Data curation, Conceptualization. **Robert Greif:** Writing – review & editing, Methodology, Formal analysis, Data curation, Conceptualization. **Cristian Abelairas-Gomez:** Writing – review & editing, Methodology, Data curation, Conceptualization. **Lucija Gosak:** Writing – review & editing, Data curation. **Yiqun Lin:** Writing – review & editing, Writing – original draft, Methodology, Formal analysis, Data curation, Conceptualization.

## Declaration of competing interest

The authors declare the following financial interests/personal relationships which may be considered as potential competing interests: YL, AL, RG, CG and AC are members of the ILCOR EIT Task Force (RG is chair, AC is vice-chair). RG is ERC Director of Guidelines and ILCOR, AL is President of Resuscitation Council UK. AC, AL, and RG are Editorial Board members of Resuscitation Plus.
