# Supplementary material for: Use of augmented and virtual reality in resuscitation training: A systematic review
Source: Resusc Plus. 2024 Apr 22;18:100643. doi: 10.1016/j.resplu.2024.100643 (PMC11053298; doi:10.1016/j.resplu.2024.100643)
Supplement: Supplementary Data 1 [file mmc1.docx]

**Online Supplementary Material**

**Database: Ovid MEDLINE(R) and Epub Ahead of Print, In-Process, In-Data-Review & Other Non-Indexed Citations and Daily**

Search Strategy:

--------------------------------------------------------------------------------

1 exp resuscitation/

2 cpr.tw,kf.

3 resuscit*.tw,kf.

4 "Cardiopulmonary resuscitation".tw,kf.

5 "Basic life support".tw,kf.

6 (basic adj4 support).tw,kf.

7 "Advanced life support".tw,kf.

8 exp Cardiopulmonary Resuscitation/

9 (Chest adj3 compress*).tw,kf.

10 "Cardiac massage".tw,kf.

11 "Cardiac life support".tw,kf.

12 "Code Blue".tw,kf.

13 exp "out of hospital cardiac arrest"/

14 exp automated external defibrillator/

15 defib*.tw,kf.

16 AED.tw,kf.

17 exp heart arrest/

18 "cardiac arrest".tw,kf.

19 exp augmented reality/

20 exp virtual reality/

21 "augmented reality".tw,kf.

22 "virtual reality".tw,kf.

23 "mixed reality".tw,kf.

24 "HTC vive".tw.

25 oculus.tw.

26 cardboard.tw.

27 hololens.tw.

28 VR.tw,kf.

29 AR.tw,kf.

30 (virtual adj4 scenario*).tw.

31 1 or 2 or 3 or 4 or 5 or 6 or 7 or 8 or 9 or 10 or 11 or 12 or 13 or 14 or 15 or 16 or 17 or 18

32 19 or 20 or 21 or 22 or 23 or 24 or 25 or 26 or 27 or 28 or 29 or 30

33 31 and 32

34 limit 33 to humans

**Database: Embase**

Search Strategy:

--------------------------------------------------------------------------------

1 exp resuscitation/

2 cpr.tw,kf.

3 resuscit*.tw,kf.

4 "Cardiopulmonary resuscitation".tw,kf.

5 "Basic life support".tw,kf.

6 (basic adj4 support).tw,kf.

7 "Advanced life support".tw,kf.

8 exp basic life support/

9 exp advanced life support/

10 (Chest adj3 compress*).tw,kf.

11 "Cardiac massage".tw,kf.

12 "Cardiac life support".tw,kf.

13 "Code Blue".tw,kf.

14 exp cardiac resynchronization therapy defibrillator/

15 exp major adverse cardiac event/

16 exp "out of hospital cardiac arrest"/

17 exp automated external defibrillator/

18 defib*.tw,kf.

19 AED.tw,kf.

20 exp heart arrest/

21 "cardiac arrest".tw,kf.

22 1 or 2 or 3 or 4 or 5 or 6 or 7 or 8 or 9 or 10 or 11 or 12 or 13 or 14 or 15 or 16 or 17 or 18 or 19 or 20 or 21

23 exp augmented reality/

24 exp virtual reality/

25 "augmented reality".tw,kf.

26 "virtual reality".tw,kf.

27 "mixed reality".tw,kf.

28 "HTC vive".tw.

29 oculus.tw.

30 cardboard.tw.

31 hololens.tw.

32 VR.tw,kf.

33 AR.tw,kf.

34 (virtual adj4 scenario*).tw.

35 23 or 24 or 25 or 26 or 27 or 28 or 29 or 30 or 31 or 32 or 33 or 34

36 22 and 35

37 compar*.tw.

38 36 and 37

39 limit 38 to human

**Database: Scopus**

Search Strategy:

("CPR Training"[All Fields] OR "cardiopulmonary resuscitation"[All Fields] OR "basic life support"[All Fields] OR "Advanced Life Support"[All Fields] OR "Chest compressions"[All Fields] OR "resuscitation"[All Fields] OR "Cardiac massage"[All Fields] OR "Cardiac life support"[All Fields] OR "Code blue"[All Fields] OR “cardiac arrest”[All Fields]) AND ("augmented reality"[All Fields] OR "virtual reality"[All Fields] OR "HTC Vive"[All Fields] OR "Oculus Rift"[All Fields] OR "Oculus Quest"[All Fields] OR "Oculus Quest 2"[All Fields] OR Cardboard[All Fields] OR "mixed reality"[All Fields] OR "hololens"[All Fields] OR "VR Sim"[All Fields] OR "VR/AR"[All Fields] OR “VR App”[All Fields] OR “Virtual scenarios“[All Fields])
